# Longitudinal Observation of Muscle Mass over 10 Years According to Serum Calcium Levels and Calcium Intake among Korean Adults Aged 50 and Older: The Korean Genome and Epidemiology Study

**DOI:** 10.3390/nu12092856

**Published:** 2020-09-18

**Authors:** Young-Sang Kim, Kyung-Won Hong, Kunhee Han, Yon Chul Park, Jae-Min Park, Kwangyoon Kim, Bom-Taeck Kim

**Affiliations:** 1 Department of Family Medicine, CHA Bundang Medical Centre, CHA University, Seongnam 13496, Korea; zeroup@cha.ac.kr; 2 Healthcare R&D Division, Theragen Bio Co. Ltd., Suwon 16229, Korea; kyungwon.hong@therabio.kr; 3 Department of Family Medicine, Seonam Hospital, Seoul 08049, Korea; hankawow@gmail.com; 4 Department of Family Medicine, Wonju Severance Christian Hospital, Wonju 26426, Korea; iamyonchul@yonsei.ac.kr; 5 Department of Medical Education, Yonsei University Wonju College of Medicine, Wonju 26426, Korea; 6 Department of Family Medicine, Gangnam Severance Hospital, Yonsei University College of Medicine, Seoul 06273, Korea; MILKCANDY@yuhs.ac; 7 Department of Family Practice and Community Health, Ajou University Hospital, Ajou University, Suwon 16499, Korea; hero8265@gmail.com

**Keywords:** calcium, muscle, serum calcium, calcium intake, muscle mass

## Abstract

The aim of this study was to investigate the longitudinal change in muscle mass over 10 years according to serum calcium levels and calcium intake. A total of 1497 men and 1845 women aged 50 years and older were included. Significant muscle loss (SML) was defined as a 5% or greater loss from baseline, while time-dependent development of SML was assessed according to quartiles for corrected calcium level and daily calcium intake using Cox regression models. The incidence of SML was 6.7 and 7.7 per 100-person-years among men and women, respectively. Groups with the lowest corrected calcium levels had more prominent SML than those with higher calcium levels, regardless of sex. The relationship between SML and calcium intake was significant only among women. The hazard ratio for SML per 1 mmol/L increase in corrected calcium level was 0.236 and 0.237 for men and women, respectively. In conclusion, low serum calcium levels may predict SML among adults aged ≥ 50 years, while low calcium intake may be a predictor for muscle loss among women. Therefore, encouraging dietary calcium intake among middle-aged and older adults for preservation of muscle mass should be considered.

## 1. Introduction

Sarcopenia is a condition characterized by age-associated muscle degeneration, which may promote reduced physical capability [1,2,3]. Sarcopenia has generally been defined as lower muscle mass and function compared to the sex-specific young reference group [4,5]. Although this operational definition has been widely used for convenience, it does not indicate individual age-associated change in muscle status. Meanwhile, studies investigating the etiology of sarcopenia have implicated various factors for its development [6], including physical activity, hormone, nutrition, inflammation, and insulin resistance.

Calcium is one of the most abundantly stored nutrients in the human body. Although most of the calcium in the body is stored in the bones, calcium is also vital for muscle function given that it binds to troponin in the muscle and allows actin and myosin to interact [7]. All muscle fibers use the calcium ion as their main regulatory and signaling molecule [8]. Given the importance of calcium for muscle function, studies have investigated the impact of calcium intake or serum levels on sarcopenia outcomes [9]. Accordingly, several studies have reported no association between calcium and muscle mass or function [10,11,12]. In contrast, studies on the Korea National Health and Nutrition Examination Survey have shown that higher calcium intake was associated with low muscle mass [13,14].

Unexpectedly, only a few studies have focused on the time-dependent decline in muscle mass. Longitudinal studies have suggested some factors, such as diabetes, smoking, and arterial stiffness, as predictive factors for accelerated skeletal muscle loss [15,16,17]. To date, however, no longitudinal study has assessed the association between calcium and muscle loss. The Korean Genome and Epidemiology Study (KoGES) is a national cohort that observes large samples of regional populations every other year [18]. Using this cohort, the present study investigated the change in muscle mass over 10 years according to calcium intake and serum calcium levels among Korean adults aged 50 years and older and determined whether the relationship was independent of muscle-associated factors.

## 2. Subjects and Methods

### 2.1. Subjects

The current study was conducted on the KoGES Ansan and Ansung cohort, which covers both rural and urban communities in South Korea. The baseline survey of the KoGES Ansan and Ansung study was completed in 2001–2002, with follow-up surveys being conducted biennially. Initially, a total of 10,038 participants aged 40–69 participated in the cohort. All subjects voluntarily participated in the study and provided informed consent.

**Figure 1:**
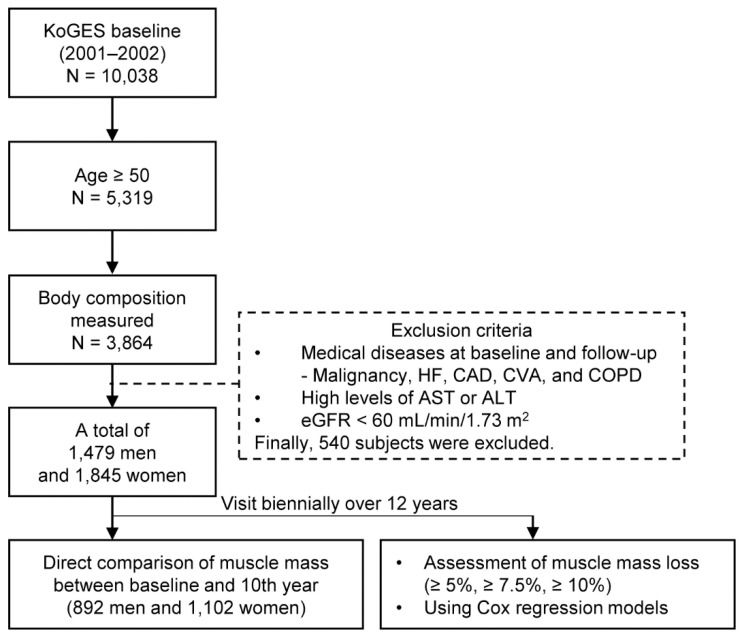
Flowchart of the study. KoGES, the Korean Genome and Epidemiology Study; HF, heart failure; CAD, coronary artery disease; CVA, cerebrovascular accident; COPD, chronic obstructive pulmonary disease; AST, Aspartate aminotransferase; ALT, Alanine aminotransferase; and eGFR, estimated glomerular filtration rate.

Given that the decrease in muscle mass accelerates at the end of the fifth decade [19], the current study identified 5319 subjects aged 50 years and older, ultimately including those whose body composition had been analyzed (*N* = 3864). Those who had suffered any type of cancer (*N* = 57), heart failure (*N* = 9), coronary artery disease (*N* = 41), cerebrovascular accident (*N* = 74), and chronic obstructive pulmonary disease (*N* = 66) were excluded. In addition, we excluded subjects with serum aspartate aminotransferase or alanine aminotransferase levels three times higher than the normal upper limits (*N* = 26) and an estimated glomerular filtration rate (GFR) < 60 mL/min/1.73 m^2^ (*N* = 132); then, we also excluded those who had developed those diseases during any time of the follow-up period (*N* = 227). Ultimately, 1479 men and 1845 women were included herein (Figure 1).

The current study has been approved by the Institutional Review Board of the CHA Bundang Medical Center in Korea (2020-02-020).

### 2.2. Medical History, Lifestyle Habits, and Anthropometric Measurements

Data regarding medical histories and lifestyle habits were obtained. Smoking and drinking status included only current smokers and excessive alcohol consumers (≥30 and ≥20 g/day for men and women), respectively. Excessive alcohol consumption was defined according to the Korean Association for the Study of the Liver. The intensities of physical activities were estimated into metabolic equivalents (MET)-h/week. Physical activity was classified into low (<15 MET-h/week), moderate, and high (≥30 MET-h/week) [20].

Height, weight, waist circumference, and blood pressure (BP) were measured in a standardized manner, while body composition was measured using body impedance analysis (BIA, ZEUS 9.9; Jawon, Daejon, Korea). To control the influence of height on body size, body weight and muscle mass were adjusted for height. Hence, body weight and muscle mass were also used to determine body mass index (BMI, body weight/height squared in kg/m^2^) and height-adjusted muscle mass (HMM, muscle mass/height squared in kg/m^2^), respectively.

### 2.3. Laboratory Tests

After fasting overnight for 12 h, plasma concentrations of glucose, lipids, protein, albumin, creatinine, and calcium were measured enzymatically using a 747 Chemistry Analyzer (Hitachi, Tokyo, Japan). Fasting plasma insulin concentrations were determined using a radioimmunoassay kit (Linco Research, St. Charles, MO, USA). Insulin resistance was approximated using the Homeostasis Model Assessment (HOMA2) calculator v2.2.3 (Oxford Center for Diabetes, Endocrinology and Metabolism, UK, available at http://www.dtu.ox.ac.uk). Corrected serum calcium was calculated using the following formula: corrected calcium (mg/dL) = serum calcium (mg/dL) + 0.8 × [4-albumin (g/dL)] [21]. Plasma renin activity (PRA) was measured using radioimmunoassay with a Cobra r-counter (PACKARD, Meriden, CT, USA), while GFR was estimated using the Modification of Diet in Renal Disease method [22].

### 2.4. Food Frequency Questionnaire (FFQ)

Dietary intake was assessed by trained dietitians using a 103-item semi-quantitative FFQ. Total calories and nutrient intakes were calculated using the standard Korean Food Composition Table.

### 2.5. Definition of Weight Loss and Muscle Loss

Body weight and muscle mass were repeatedly measured at each visit, from which the rate of change was subsequently calculated. Significant weight loss was defined as a >5% reduction in body weight compared to the baseline [23]. Similarly, significant muscle mass loss was defined as a ≥5% muscle loss compared to the baseline. Considering that this definition indicated weight loss for over a year or less [23], further analysis was conducted utilizing 7.5% and 10% to indicate significant weight and muscle loss instead of 5%.

### 2.6. Statistical Analysis

For descriptive analysis, results were expressed as mean ±SD, median (interquartile range), or number (proportion). The independent t-test, Mann–Whitney U test, and chi-square test were used to compare variables between men and women. To reduce skewness, some variables, such as PRA, triglycerides, and H2IR, were used in the subsequent parametric analyses after logarithmical transformation. Considering differences in body composition, laboratory results, and lifestyle, subsequent sex-specific analyses were performed.

To assess differences in body weight and muscle mass according to calcium status, mean body weight, BMI, muscle mass, and HMM were compared according to the quartiles of corrected calcium levels and calcium intake using analysis of variance (ANOVA). Spearman correlation analyses were used to determine whether laboratory results and FFQ were associated with body weight and muscle mass.

Longitudinal change in body weight and muscle mass was evaluated. First, the rate of change in body weight and muscle mass according to quartiles of bassline serum calcium level and calcium intake was compared between the first and 10th year using ANOVA. Second, the development of the significant weight loss and muscle mass loss according to the quartiles was assessed. Kaplan–Meier curves for significant weight loss and muscle mass loss were created for the quartiles of corrected calcium levels and calcium intake, after which the curves were compared using the log-rank test. To calculate the hazard ratio (HR) of the quartiles for the significant muscle mass loss, stepwise Cox regression models were formulated with potential confounders selected based on previous correlation analyses. The first adjusted model (Model 2) included covariates associated with general information and lifestyle habits, such as age, height, energy and protein intake, physical activity, smoking, and alcohol consumption. Model 3 additionally included variables related to the renin-angiotensin system and metabolic parameters, such as PRA, GFR, BP, and high-density lipoprotein cholesterol. Model 4 was additionally adjusted for medical history of hypertension and diabetes, while Model 5 was additionally adjusted for baseline body weight or muscle mass. The HRs per unit change in corrected calcium levels was also calculated using Cox regression analysis with the same covariates used in Model 5. After changing the definitions for significant loss to 7.5% and 10%, Cox models were reformulated. To reduce the influence of the extreme values of baseline muscle mass, another Cox regression model trimmed the upper and lower 10% of the subjects. After trimming the subjects out of the serum calcium reference range, another Cox regression model was formulated. All statistical analyses were performed using SPSS 26.0 (IBM, Armonk, NY, USA), with *P* < 0.05 indicating statistical significance.

## 3. Results

Table 1 summarizes the baseline characteristics of all included participants. Accordingly, men and women had a mean age of 58.7 and 59.1 years, respectively. BMI was higher among women (25.2 kg/m^2^) than men (23.9 kg/m^2^), whereas HMM was higher among men than women. Mean serum calcium concentrations did not differ significantly between sexes (2.41 mmol/L). Mean corrected calcium concentrations were 2.37 and 2.38 mmol/L in men and women, respectively. According to the FFQ, men had a higher daily energy and macronutrient intake than women. Mean calcium intake was 468.5 ± 250.9 and 462.4 ± 266.8 mg/d in men and women, respectively.

Serum corrected calcium concentrations and daily calcium intake were categorized into quartiles according to sex (Table S1). The ranges of corrected calcium were 1.93–2.31, 2.32–2.39, 2.40–2.46, and 2.46–2.94 mmol/L in Q1 to Q4 in women. The ranges of calcium intake were 18.3–279.7, 279.9–406.6, 407.4–574.0, and over 574.5 mg/day in women. The ranges in men were similar to those in women (Table S1). Men exhibited a decrease in HMM as the quartile of corrected calcium increased, whereas no such difference was observed among women. BMI and HMM were lowest in the first quartile of calcium intake among men. Similarly, body weight and muscle mass were lowest in Q1 among women.

**Figure 2:**
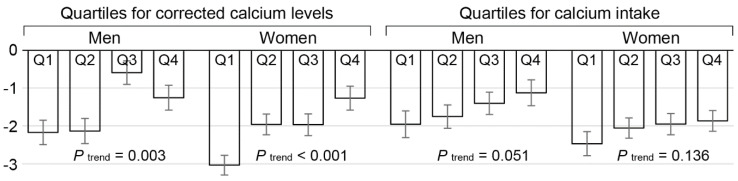
Muscle mass change from baseline to 10th year according to quartiles for corrected calcium levels and daily calcium intake. Decreased muscle mass was observed as quartiles for corrected calcium levels escalated. In contrast, no significant difference in muscle loss was observed according to quartiles for calcium intake in both men and women. Error bars represent SEM.

The time-associated rate of muscle mass change over 10 years is presented in Figure 2. The rate of muscle mass change from the baseline to the 10th year was compared among the quartiles. The mean rate of change differed significantly among the quartiles of corrected calcium levels in both men and women (*P* for trend = 0.003 and <0.001 in men and women, respectively). However, no significant difference in the rate of change was observed among the quartiles of calcium intake in either men or women.

**Figure 3:**
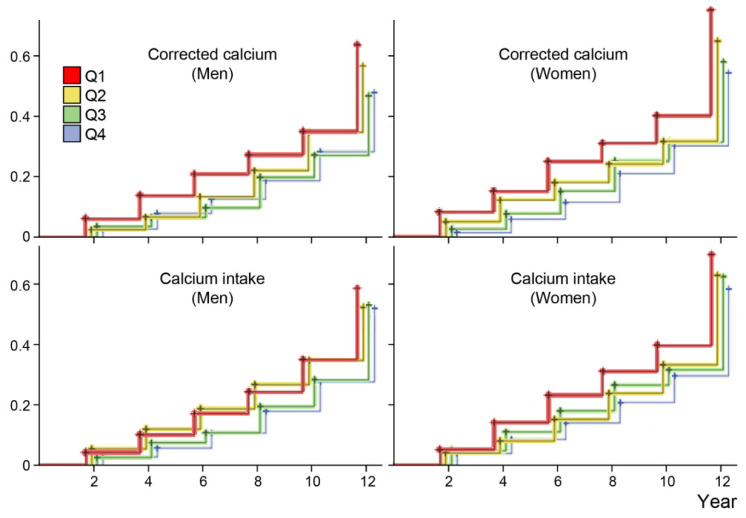
Cumulative incidence of significant muscle mass loss according to quartiles for corrected calcium level and daily calcium intake. Lower corrected calcium levels were significantly associated with loss of muscle mass (*P* < 0.001 using the log-rank test in both men and women). Daily calcium intake was associated with loss of muscle mass only among women (*P* = 0.004), not men (*P* = 0.124).

The incidence (cumulative method) and incidence density rates for significant muscle loss were 42.4 per 100 persons and 6.7 per 100 person-years in men and 48.1 per 100 persons and 7.7 per 100 person-years in women, respectively (Table S2). Table S2 outlines the incidences of significant muscle loss according to established loss rates. The time-related muscle loss (≥5%) was expressed in Kaplan–Meier curves according to the quartiles of corrected calcium levels and calcium intake (Figure 3). Accordingly, the curves differed significantly among the quartiles of corrected calcium levels in both men and women (*P* < 0.001 estimated using the log-rank test). However, the difference in calcium intake was significant only among women, not men.

**Figure 4:**
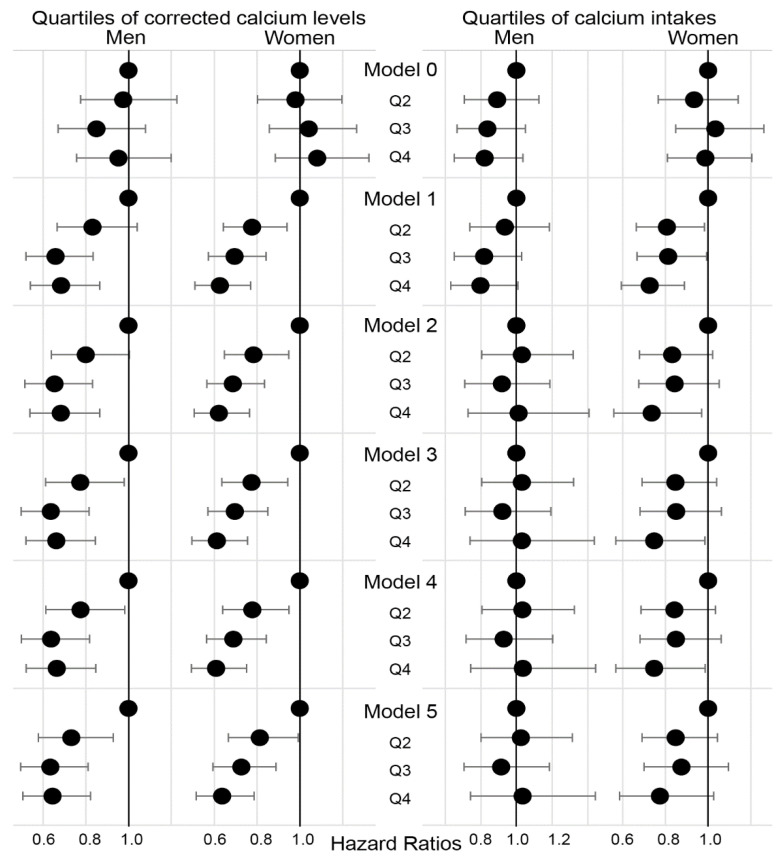
Hazard ratios for quartiles of corrected calcium levels and daily calcium intake. Models 0 and 1 are crude models for weight loss and muscle loss, respectively. Model 2 is a model for muscle loss adjusted for age, height, energy and protein intake, and lifestyle habits. Model 3 additionally adjusts for variables related to renin-angiotensin system and metabolic parameters. Model 4 additionally adjusts for history of hypertension and diabetes. Model 5 additionally adjusts for baseline muscle mass.

The risk for significant weight (5%) and muscle (5%) loss was assessed using Cox regression models (Figure 4). The HRs for Q2, Q3, and Q4 were calculated relative to Q1. In Model 0, no significant weight loss was observed among the quartiles of corrected calcium levels and calcium intake. Significant muscle loss was less apparent in Q2–Q4 than in Q1 of corrected calcium levels in both men and women. Adjustment for potential confounders did not influence the longitudinal relationship between corrected calcium levels and the development of significant muscle loss (Models 2–5). The HRs for significant body weight and muscle loss were reassessed per 1 mmol/L increase in calcium level as a continuous variable (Table S3). Accordingly, the HR for 5% muscle mass loss per 1 mmol/L increase in corrected calcium levels was 0.236 and 0.237 in men and women, respectively (Model B). Using measured calcium levels instead of corrected calcium did not change the significance of the model (Model C). When 7.5% or 10% loss was utilized instead of 5%, the significance of the models remained unchanged (Models D and E). Moreover, excluding subjects beyond the reference range of serum calcium or those with extreme baseline muscle mass values did not affect the significance of the model (Models F and G).

## 4. Discussion

The current study investigated the longitudinal relationship between calcium and muscle loss over a maximum of 12 years. Accordingly, our results showed that low serum calcium levels significantly predicted muscle loss but not weight loss. Moreover, the ability of low calcium intake to predict muscle mass loss was significant only among women.

Given that most of the calcium is stored in mineralized tissues, the majority of studies have focused on the relationship between calcium and bone. Several studies have reported that calcium intake or supplements increase bone mineral density and reduce fracture [24,25], while others found no association between calcium intake and postmenopausal bone loss or risk of fracture [26,27]. Moreover, studies investigating the association between calcium and fat tissue and obesity have found that dietary calcium intake was associated with anti-obesity effects [28,29,30].

Only a few studies have described the relationship between calcium intake and muscle mass or function. Accordingly, studies have found no association between calcium intake and sarcopenia defined using the combination of muscle mass, grip strength, and muscle function [10,11]. Despite the absence of data on muscle strength and function, the current study described muscle mass according to calcium intake. No difference in HMM was observed among Q2–Q4 in men and Q1–Q4 in women. In contrast, a Korean study reported that individuals with low calcium intake had decreased muscle mass and increased fat proportions [14]. Korean cross-sectional studies on the relationship between calcium intake and muscle mass utilized the definition of low muscle mass that adjusted for body weight [13,14]. Given that a weight-adjusted definition may not distinguish low muscle mass from adiposity, interpreting the results of such studies may be difficult. Another study compared serum calcium levels between subjects with normal muscle mass and low muscle mass defined using various methods [31], subsequently finding no difference in calcium levels between the subjects regardless of definitions.

No previous longitudinal studies have investigated the age-related changes in muscle mass according to serum calcium levels or calcium intake. Our study was the first to show that higher loss of muscle mass was related to lower levels of baseline serum calcium levels among both men and women and lower calcium intake in women. Our results suggest that relative calcium insufficiency may accelerate muscle mass loss. Calcium is a key mineral that regulates muscle contraction and nerve impulse conduction [32]. Basically, calcium insufficiency may lead to muscle dystrophy given its influence on muscle function. Hypercalcemia may also negatively affect the musculoskeletal system. However, the current study included only a small number of subjects that had serum calcium levels beyond the reference range, while results remained unchanged even after excluding such subjects. Although vitamin D levels were not measured herein, vitamin D may also influence both calcium levels and skeletal muscle. Considering that vitamin D regulates calcium homeostasis, 1,25-dihydroxyvitamin D3-induced calcium flux and alteration in calcium signaling may play a role in regulating muscle contractile force in differentiated muscle fibers [33]. It is also notable that the decline in muscle mass and function with age is concurrent with a decline in skeletal muscle vitamin D receptor expression [34].

The amount of calcium intake in our population is problematic, with a median intake of approximately 400–420 mg/day, similar to that presented in the Korea National Health and Nutrition Examination Survey (KNHANES) [35]. However, these amounts are considerably lower than that recommended by the Korean Nutrition Society (700 mg) or National Institutes of Health (1000–1200 mg) [36,37], with only 3.8% of our population having a calcium intake of more than 1000 mg/day (data not shown). Several studies have warned of the adverse effects of a high calcium load. Higher circulating calcium levels have been associated with an increased risk of type 2 diabetes [38], while calcium supplements have been associated with an increased risk of myocardial infarction [39]. Nonetheless, given the much lower calcium intake in our population relative to most of the referred studies and the negative effects of lower serum calcium levels on muscle preservation, it may be reasonable to encourage increased calcium intake.

Our study had several limitations worth noting. First, muscle mass was estimated using the BIA method. While dual-energy X-ray absorptiometry (DXA) has been preferred as a standard method, appendicular muscle mass measured using DXA has been commonly used to define sarcopenia [40,41]. However, BIA has also been regarded as a good method to measure skeletal muscle mass [5], given that it has been better validated compared to magnetic resonance imaging [42]. Second, while sarcopenia is defined as a combination of decreased muscle mass, strength, and function [40,41], the current study did not investigate muscle strength and function. Further studies need to investigate changes in muscle strength and function aside from muscle mass. Third, some factors determining circulating calcium concentrations were not included in the current study. In particular, vitamin D and parathyroid hormone can influence body calcium, including serum concentrations. Considering the relationship between 25-hydroxyvitamin D and low muscle mass [43,44], vitamin D may play a role in the link between calcium and muscle mass. Fourth, FFQ was not repeated on every visit. Eating habit change may influence body composition. Lastly, FFQ did not collect information on calcium supplementation, although physiological studies have suggested no material differences in the metabolic actions of dietary calcium and that obtained from supplements [45]. However, given that supplementary calcium intake may cause temporary hypercalcemia [46], the functional role between dietary and supplementary calcium may differ. An additional survey of calcium supplementation may enrich our analyses.

## 5. Conclusions

Low serum calcium levels may predict muscle loss among Korean adults aged 50 years and older over a maximum of 12 years. Low dietary calcium intake may also predict accelerated muscle loss among women. Therefore, recommending increased dietary calcium intake may be worthwhile for the preservation of muscle mass. Nonetheless, further studies are required to assess the influence of calcium on changes in muscle strength and function.

## Figures and Tables

**Table 1:** Baseline characteristics of the study subjects.

	Men (*N* = 1479)	Women (*N* = 1845)	*P*
Age (years)	58.7 ± 5.7	59.1 ± 5.6	0.040
Current smoker	657 (44.4%)	60 (3.3%)	<0.001
Alcohol consumer	235 (15.9%)	17 (0.9%)	<0.001
Physical activity			
Mild	450 (30.4%)	669 (36.3%)	<0.001
Moderate	281 (19.0%)	411 (22.3%)	
High	748 (50.6%)	765 (41.5%)	
Hypertension	228 (15.4%)	419 (22.7%)	<0.001
Diabetes	153 (10.3%)	153 (8.3%)	0.048
Anthropometric measurement and body composition			
Height (cm)	165.1 ± 5.8	152.4 ± 5.4	<0.001
Body weight (kg)	65.3 ± 9.7	58.6 ± 8.7	<0.001
Body mass index (kg/m^2^)	23.9 ± 2.9	25.2 ± 3.3	<0.001
Muscle mass (kg)	47.9 ± 6.0	36.8 ± 4.2	<0.001
Muscle/height^2^ (kg/m^2^)	18.5 ± 1.6	16.8 ± 1.3	<0.001
Metabolic parameters and laboratory results			
Systolic BP (mmHg)	125.6 ± 17.8	126.6 ± 19.6	0.154
Diastolic BP (mmHg)	82.5 ± 10.6	81.2 ± 11.3	<0.001
Glucose (mmol/L)	4.94 (4.61–5.44)	4.83 (4.55–5.22)	<0.001
Insulin (pmol/L)	42.4 (32.6–58.3)	50.0 (37.3–68.2)	<0.001
HOMA2-IR	0.80 (0.61–1.11)	0.94 (0.69–1.27)	<0.001
Total cholesterol (mmol/L)	5.02 ± 0.93	5.37 ± 0.98	<0.001
HDL cholesterol (mmol/L)	1.25 ± 0.31	1.30 ± 0.31	<0.001
Triglyceride (mmol/L)	1.51 (1.04–2.24)	1.47 (1.06–2.07)	0.114
Plasma renin activity (ng/mL/h)	2.15 (1.09–3.80)	1.21 (0.57–2.38)	<0.001
eGFR (mL/min/1.73 m^2^)	94.1 ± 18.4	87.3 ± 13.0	<0.001
Total protein (mmol/L)	72.8 ± 4.5	72.7 ± 4.1	0.740
Albumin (mmol/L)	45.2 ± 3.0	44.4 ± 2.5	<0.001
Calcium (mmol/L)	2.41 ± 0.12	2.41 ± 0.11	0.058
Corrected calcium (mmol/L)	2.37 ± 0.13	2.38 ± 0.12	0.080
Food frequency questionnaire			
Energy (kcal/d)	2006.3 ± 690.7	1833.7 ± 664.8	<0.001
Carbohydrate (g/d)	350.9 ± 111.0	336.0 ± 123.3	<0.001
Protein (g/d)	68.4 ± 31.3	60.2 ± 25.8	<0.001
Fat (g/d)	33.9 ± 22.5	25.6 ± 15.3	<0.001
Calcium (mg/d)	468.5 ± 250.9	462.4 ± 266.8	0.505

Data are expressed as mean ±SD, median (interquartile range), or number (proportion). BP, blood pressure; HOMA2-IR, homeostatic model assessment for insulin resistance; HDL, high-density lipoprotein; and eGFR, estimated glomerular filtration rate.

## Supplementary Materials

The following are available online at https://www.mdpi.com/2072-6643/12/9/2856/s1: Table S1: Body weight and muscle mass according to quartiles of serum calcium levels and daily calcium intake at baseline; Table S2: Incidence of significant loss of body weight and muscle mass according to definitions; and Table S3: Hazard ratios per unit increase in serum calcium concentration for each end point.
