# Supplementary material for: Longitudinal Observation of Muscle Mass over 10 Years According to Serum Calcium Levels and Calcium Intake among Korean Adults Aged 50 and Older: The Korean Genome and Epidemiology Study
Source: Nutrients. 2020 Sep 18;12(9):2856. doi: 10.3390/nu12092856 (PMC7551872; doi:10.3390/nu12092856)
Supplement: Supplementary file 1 [file nutrients-12-02856-s001.pdf]

Table S1. Body weight and muscle mass according to quartiles of serum calcium levels and daily calcium intake at baseline.

	Men					Women				
	**Range**	Weight	BMI	Muscle mass	**HMM**	**Range**	**Weight**	**BMI**	**Muscle mass**	**HMM**
Corrected calcium (mmol/L)										
Q1	1.91–2.30	63.1 ± 9.5	23.8 ± 2.9	48.2 ± 6.0	18.6 ± 1.6	1.93–2.31	58.0 ± 8.7	24.9 ± 3.2	37.1 ± 4.3	16.9 ± 1.3
Q2	2.30–2.38	65.7 ± 9.5	24.0 ± 2.9	47.9 ± 5.7	18.6 ± 1.5	2.32–2.39	58.4 ± 8.7	25.2 ± 3.4	36.6 ± 4.2	16.8 ± 1.3
Q3	2.39–2.45	66.0 ± 9.9	23.8 ± 3.0	47.6 ± 6.3	18.4 ± 1.6	2.40–2.46	58.8 ± 8.6	25.2 ± 3.2	36.9 ± 4.3	16.8 ± 1.4
Q4	2.46–2.74	65.4 ± 9.7	24.0 ± 3.0	47.7 ± 5.8	18.5 ± 1.6	2.46–2.94	59.0 ± 8.7	25.4 ± 3.2	36.6 ± 4.3	16.7 ± 1.3
P trend		0.735	0.788	0.197	0.047		0.054	0.026	0.213	0.086
Calcium intake (mg/day)										
Q1	68.0–298.0	63.1 ± 9.5	23.4 ± 2.9	46.5 ± 5.9	18.2 ± 1.6	18.3–279.7	58.0 ± 9.4	25.2 ± 3.5	36.5 ± 4.6	16.8 ± 1.5
Q2	298.4–422.3	65.7 ± 9.5	24.1 ± 3.0	48.1 ± 5.7	18.6 ± 1.6	279.9–406.6	58.2 ± 8.2	25.1 ± 3.2	36.6 ± 4.0	16.8 ± 1.3
Q3	422.3–579.8	66.0 ± 9.9	24.1 ± 2.9	48.2 ± 6.0	18.6 ± 1.6	407.4–574.0	58.4 ± 8.2	25.1 ± 3.1	36.7 ± 4.1	16.7 ± 1.3
Q4	579.9–2309.7	66.4 ± 9.7	24.1 ± 2.9	48.6 ± 5.9	18.6 ± 1.5	574.5–2986.5	59.7 ± 8.7	25.4 ± 3.2	37.4 ± 4.2	16.9 ± 1.3
P trend		<0.001	0.003	<0.001	0.003		0.003	0.419	0.001	0.640

Data are expressed as mean ± SD. BMI, body mass index; HMM, height-adjusted muscle mass (kg/m^2)

Table S2. Incidence of significant loss of body weight and muscle mass according to definitions.

	Weight Loss		Muscle Mass Loss	
	**Cumulative Method (per 100 persons)**	**Incidence Density (per 100-person-years)**	**Cumulative Method (per 100 persons)**	**Incidence Density (per 100-person-years)**
Men				
5%	42.3	9.8	42.4	6.7
7.5%	24.5	4.8	22.9	2.8
10%	13.0	2.0	9.4	1.1
Women				
5%	48.8	11.3	48.1	7.7
7.5%	31.4	6.0	32.4	4.3
10%	19.1	3.2	18.9	2.1

Table S3. Hazard ratios per unit increase in serum calcium concentration for each end point.

		Men		Women	
**Model**	**End Point**	**Hazard Ratio**	***P***	**Hazard Ratio**	***P***
Model A	Weight loss (5%)	0.830 (0.430–1.604)	0.580	1.321 (0.715–2.441)	0.374
Model B	Muscle loss (5%)	0.236 (0.121–0.461)	<0.001	0.237 (0.130–0.430)	<0.001
Model C	Muscle loss (5%)	0.211 (0.101–0.441)	<0.001	0.255 (0.130–0.501)	<0.001
Model D	Muscle loss (7.5%)	0.163 (0.066–0.401)	<0.001	0.208 (0.103–0.418)	<0.001
Model E	Muscle loss (10%)	0.137 (0.032–0.585)	0.007	0.199 (0.079–0.498)	0.001
Model F	Muscle loss (5%)	0.205 (0.094–0.445)	<0.001	0.217 (0.113–0.418)	<0.001
Model G	Muscle loss (5%)	0.266 (0.125–0.567)	0.001	0.197 (0.100–0.389)	<0.001

Hazard ratios are risk ratios for 1 mmol/L increase in corrected calcium for the end point. Measured serum calcium level was used instead of corrected calcium only in Model C. In Model F, subjects who were not within the reference for serum calcium were excluded (149 men and 136 women). In Model G, subjects who were in the upper and the lower 10% for sex-specific distribution of muscle mass were excluded.
